# Field propagation-induced directionality of carrier-envelope phase-controlled photoemission from nanospheres

**DOI:** 10.1038/ncomms8944

**Published:** 2015-08-12

**Authors:** F. Süßmann, L. Seiffert, S. Zherebtsov, V. Mondes, J. Stierle, M. Arbeiter, J. Plenge, P. Rupp, C. Peltz, A. Kessel, S. A. Trushin, B. Ahn, D. Kim, C. Graf, E. Rühl, M. F. Kling, T. Fennel

**Affiliations:** 1 Max-Planck-Institut für Quantenoptik, D-85748 Garching, Germany; 2 Physics Department, Ludwig-Maximilians-Universität München, D-85748 Garching, Germany; 3 Institut für Physik, Universität Rostock, D-18051 Rostock, Germany; 4 Physical Chemistry, Freie Universität Berlin, Takustr. 3, D-14195 Berlin, Germany; 5 Department of Physics, Center for Attosecond Science and Technology, Pohang University of Science and Technology, Pohang 790-784, South Korea; 6 Max Planck Center for Attosecond Science, Max Planck POSTECH/KOREA Res. Init., Pohang 790-784, South Korea; 7 J.R. Macdonald Laboratory, Physics Department, Kansas-State University, Manhattan, Kansas, USA

## Abstract

Near-fields of non-resonantly laser-excited nanostructures enable strong localization of ultrashort light fields and have opened novel routes to fundamentally modify and control electronic strong-field processes. Harnessing spatiotemporally tunable near-fields for the steering of sub-cycle electron dynamics may enable ultrafast optoelectronic devices and unprecedented control in the generation of attosecond electron and photon pulses. Here we utilize unsupported sub-wavelength dielectric nanospheres to generate near-fields with adjustable structure and study the resulting strong-field dynamics via photoelectron imaging. We demonstrate field propagation-induced tunability of the emission direction of fast recollision electrons up to a regime, where nonlinear charge interaction effects become dominant in the acceleration process. Our analysis supports that the timing of the recollision process remains controllable with attosecond resolution by the carrier-envelope phase, indicating the possibility to expand near-field-mediated control far into the realm of high-field phenomena.

## Abstract (web summary)

The localized enhancement of laser light in optical near-fields of nanostructures enables the steering of ultrafast electronic motion. Here, the authors employ field propagation in nanospheres to obtain directional tunability and attosecond control of near-field-induced strong-field photoemission.

## Graphical abstract

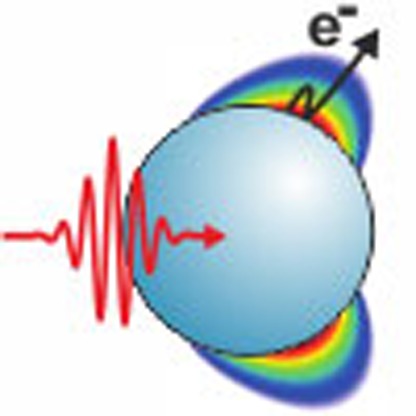

Nanostructures enable the concentration of laser light in highly localized near-fields with dimensions far below the incident wavelength^1^. Utilizing optical near-fields for the control of electron motion in nanostructures with attosecond resolution is a major prospect and challenge in ultrafast light-wave driven nanoelectronics^2^. Enhanced strong-field photoemission in near-fields has been demonstrated for metal nanotips^3^,^4^,^5^,^6^,^7^, dielectric nanospheres^8^,^9^ and surface-assembled nanoantennas^10^. In analogy to atomic above-threshold ionization, electron backscattering dominates the high-energy electron emission if the electron quiver amplitude is small compared with the near-field extension into free space. Many-particle charge interaction effects can increase the electron cutoff energy far beyond the values expected from the linear field enhancement, as demonstrated in previous experiments on small nanospheres^8^,^9^. The coherent nature of the near-field driven acceleration has been revealed by the fact that photoelectron spectra depend on the laser's electric field waveform, controlled by the carrier-envelope phase (CEP)^4^,^5^,^8^,^9^.

Key prerequisite for near-field-mediated tailoring of the underlying electronic strong-field dynamics is knowledge about its dependence on and feedback to the spatiotemporal near-field evolution. A detailed exploration of near-field control up to high field intensities is offered by studying unsupported reproducible nanosystems in the gas phase. Theoretical studies of the electron acceleration from droplets support that propagation-induced near-field effects have a strong impact on the electron dynamics, including the directionality of the emission, and can be utilized for the generation of attosecond electron bunches up to relativistic intensities^11^,^12^. Control of photoelectron angular distributions has also been demonstrated for atoms^13^ and chiral molecules^14^ via polarization-shaped femtosecond laser pulses. In the latter cases, however, the directionality results from selection rules and the chirality of the electronic initial or/and continuum states, respectively, and can be described in dipole approximation. The physics is therefore fundamentally different from control of electronic motion via field propagation-induced near-fields as considered in the present work.

Here we employ isolated 50–550 nm SiO_2_ nanospheres (Fig. 1a) and demonstrate the size-dependent effect of field propagation-induced near-field deformation on the directionality of the strong-field photoemission. We observe systematic directional tunability of the electron emission with respect to the propagation direction via the sphere size and find evidence for the persistence of robust attosecond control of the dominant surface backscattering process via the CEP. Our combined experimental and theoretical analysis shows that dynamical many-particle charge interaction results in substantial quenching of the electron emission and becomes dominant for the electron acceleration for the largest investigated sphere sizes. A systematic trajectory analysis based on semiclassical transport simulations enables for a clear discrimination of the impact of near-field enhancement, vectorial field properties and self-consistent collective electron dynamics on the strong-field photoemission process.

## Results

### Propagation-induced near field deformation

**Figure 1:**
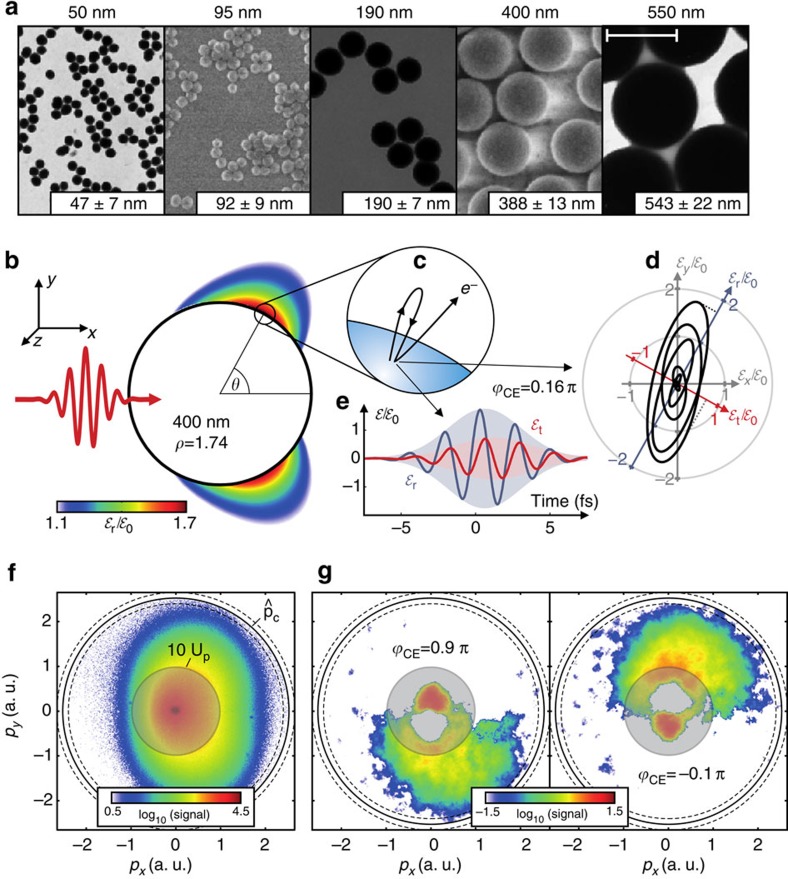
Photoemission from large nanospheres. (**a**) Typical electron microscopy images of the employed silica nanospheres. The indicated diameters (top) reflect typical sizes and are used as a reference throughout the manuscript. Measured mean diameters and their s.d. of representative samples studied in this work are indicated in boxes, respectively. The scale bar applies to all images and corresponds to a length of 500 nm. (**b**) Maximum enhancement of the radial electric field in the propagation plane (*z*=0) predicted by Mie's solution for *d*=400 nm (Mie size parameter *ρ* as indicated). The incident laser field 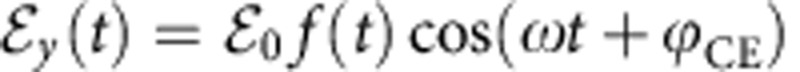 with 4 fs (intensity full-width at half-maximum) Gaussian envelope *f*(*t*) at centre wavelength *λ*=720 nm propagates along the *x* axis. (**c**) Schematic illustration of the electron recollision process. (**d**) Vectorial representation of the field evolution in the *x*−*y* plane normalized to incident peak amplitude 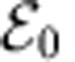, sampled at the point with peak radial near-field enhancement (*θ*=61.0°, CEP as indicated). Coloured arrows indicate the local reference frame for radial (red) and tangential (blue) fields to illustrate the evolution of the field ellipticity. (**e**) Evolution of radial and tangential electric field components 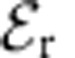 (blue) and 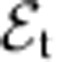 (red). (**f**) CEP-averaged VMI electron momentum projection (momenta in atomic units) measured for *d*=400 nm at 2.7 × 10^13^ W cm^−2^. (**g**) Phase-resolved VMI images (CEP as indicated) after subtraction of the CEP-averaged spectra. Solid circles in (**f**) and (**g**) indicate the extracted cutoff momentum and dashed circles the uncertainty estimated from the deviation of the results for the upper and lower half of the momentum distribution. The shaded circular areas in (**f**, **g**) indicate the momentum range that corresponds to electron energies below the 10 *U*_p_ classical rescattering cutoff and may contain residual signal from background gas.

Exposing nanospheres to few-cycle pulses with known CEP allows the generation of well-defined near-fields, whose linear response structure is accurately described by the Mie solution^15^. For a given refractive index, the latter depends mainly on the dimensionless Mie size parameter *ρ*=*πd*/*λ* (where *d* is the sphere diameter and *λ* is the wavelength) and resembles Rayleigh's quasi-static dipole solution^16^ for small spheres (*ρ*≪1). Propagation-induced near-field deformation arises and becomes significant for *ρ*≳1 because of substantial excitation of higher order multipole modes^15^, resulting in a gradual shift of the region of maximal field enhancement in propagation direction (Fig. 1b). Increasing the size parameter to the range *ρ*≫1 leads first to nanojet-type focusing employed in superlenses^17^,^18^ followed by the regime of geometric optics^19^. Such systematic modifications can be achieved by changing the excitation wavelength or the sphere size. We varied the sphere size to realize scale parameters between *ρ*≈0.2 and 2.4, ensuring peak field enhancement to occur at the surface; the employed wide-bandgap dielectric material ensures minimal pulse broadening such that the few-cycle character of the near-field is preserved. Nevertheless, field propagation induces a nontrivial elliptic local field (see Fig. 1d,e and the Methods).

### Size- and carrier-envelope phase-dependent directionality

We measured the angle-resolved photoemission from SiO_2_ nanospheres (*d*≈50−550 nm, cf. Fig. 1a) via velocity-map imaging (VMI) using a setup similar to Zherebtsov *et al.*^9^. The photoelectron dynamics was controlled by the CEP, 
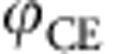
, of 4 fs few-cycle laser fields at 720 nm central wavelength (see Methods). Near-field induced symmetry breaking of the photoemission for *ρ*≳1 is revealed by the asymmetry of CEP-averaged photoelectron momentum projections with respect to the laser propagation direction (left-to-right in Fig. 1f). Below the 10 *U*_p_ backscattering cutoff (see shaded areas in Fig. 1f,g), the momentum maps may contain spurious photoemission signal from residual background gas. The CEP-dependent signal from nanospheres shows that the electron emission can be effectively switched upwards or downwards while the left-right asymmetry remains (Fig. 1g). From the modulus of the projected momentum up to which phase-dependent VMI signal is observed we determined the global cutoff momentum 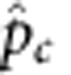 (solid circles) to define the cutoff energy 
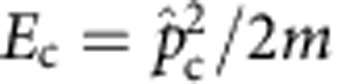
, where *m* is the electron mass. This cutoff is attributed to surface backscattering, see Fig. 1c.

**Figure 2:**
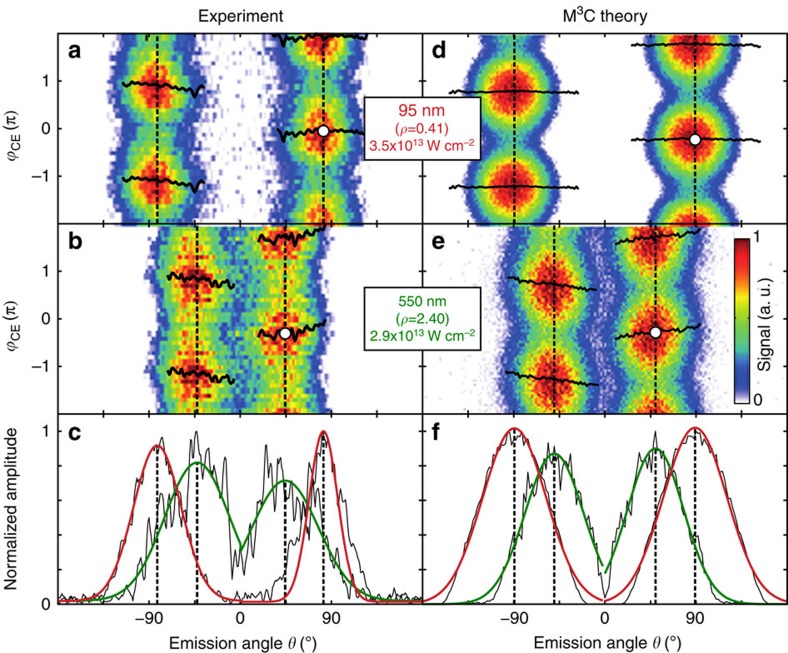
Directionality and phase-dependent switching. (**a**,**b**) Measured angle and CEP-resolved electron yields 
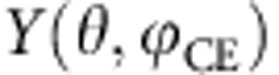
 of near-cutoff electrons (projected momenta 
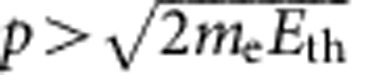
 with *E*_th_=0.5*E*_c_, discriminating electrons below the threshold energy *E*_th_; sphere sizes and laser intensities as indicated. (**c**) Amplitudes *A*(*θ*) of harmonic fits (black) of the data in (**a**,**b**) with 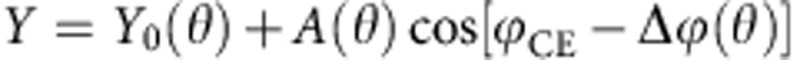 and critical emission angles (vertical dashed lines) as determined from the peaks of Gaussian fits of *A*(*θ*) (green and red curves) for upward and downward emission, respectively. Horizontal black curves in (**a**,**b**) show the fitted phase offsets 
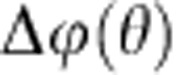
; white dots indicate the critical CEP values 
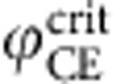
 and critical emission angles 
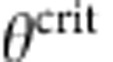
 for maximal upward emission. (**d**–**f**) same as (**a**–**c**) as predicted from M^3^C simulations for the experimental parameters.

Most importantly, we observed a size-dependent directionality of the phase-controlled photoemission. Maps of the high-energy electron yield show strong phase dynamics and signal concentration in a narrow angular range for the upward and downward direction for all investigated sphere diameters, see examples in Fig. 2a,b. From each map we extracted the critical final emission angle, 
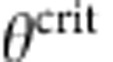
, and critical CEP, 
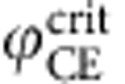
, to characterize the directionality and CEP dependence (see circular symbols). Comparison of results for 95 and 550 nm spheres reveals similar phase dynamics but a significant shift of the critical emission directions from nearly 90° to almost 45°.

### Simple man's model

**Figure 3:**
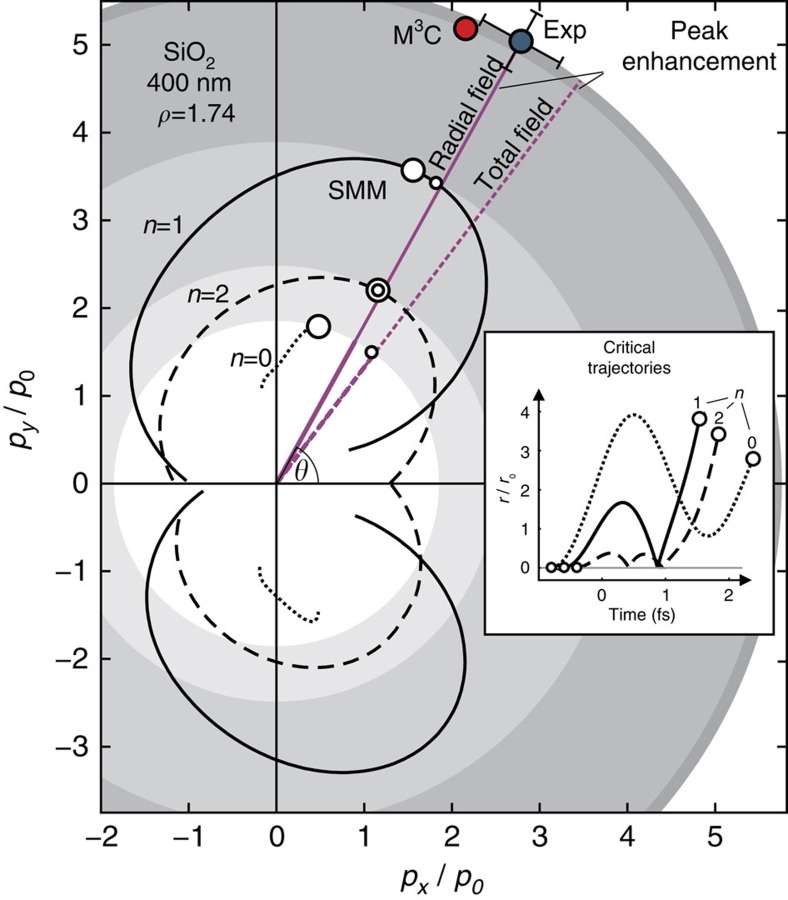
Photoemission dynamics predicted by simple man's model. Predicted electron cutoff momenta as function of emission angle, *p*_c_(*θ*_f_), for *n*=0, 1, 2 recollisions at optimal CEP for upward emission; 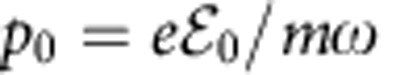 is the free space quiver momentum. Small/large circular symbols denote the critical birth/emission angles associated with respective highest cutoff momenta (see inset for corresponding radial trajectories); blue/red filled circles show critical emission angles and cutoff momenta from experiment and M^3^C simulations; shaded rings are guides to the eye.

To explore the impact of the linear near-field we describe the strong-field photoemission dynamics classically^20^ with the simple man's model (SMM). Electron trajectories launched at rest at the nanosphere surface are integrated under the local field obtained from Mie's solution and assuming elastic specular reflection at the surface. The resulting cutoff momenta as function of the asymptotic emission angle, *p*_c_(*θ*), indicate maximal energies for single recollision trajectories (*n*=1) and substantially lower energies for direct (*n*=0) and higher order (*n*>1) recollision electrons (Fig. 3). The critical birth angle of most energetic electrons (*n*=1, small circle) coincides with the angle of peak radial near-field enhancement and deviates only weakly from the critical final angle (large circle), underlining the dominance of the radial field in the recollision-based process. Comparison with experiment shows that the SMM reasonably explains the critical emission angles but substantially underestimates the observed global cutoff momenta (blue filled circle).

### Self-consistent simulation model

**Figure 4:**
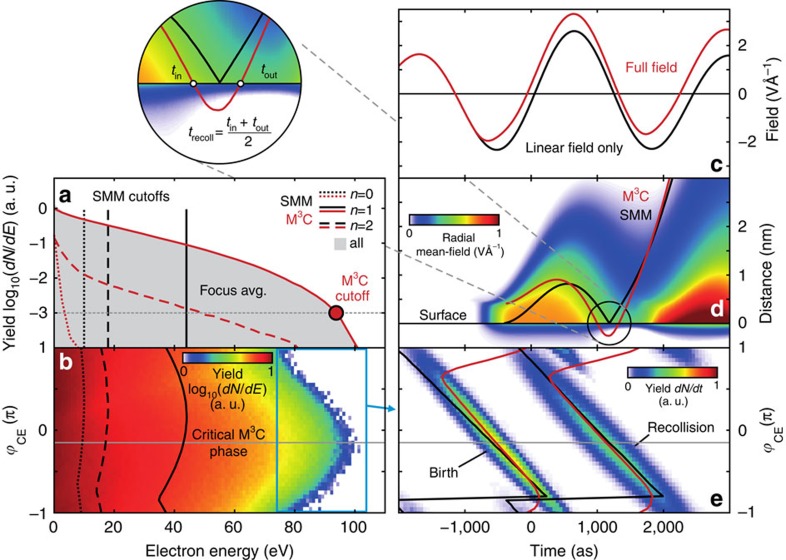
Electron energy spectra and recollision dynamics calculated with M^3^C simulations. (**a**) Recollision-resolved M^3^C energy spectra (CEP-averaged, *I*=3 × 10^13^ W cm^−2^) and respective SMM cutoffs for direct and rescattering electrons for 400 nm spheres. The index *n* indicates the number of recollisions with the sphere. The M^3^C cutoff (red circular symbol) is assigned to the energy where the spectrum of single recollision electrons (solid red curve) has dropped by a factor of 10^3^. (**b**) CEP-resolved M^3^C energy spectra (*n*=1, colour coded) for the same parameters as in (**a**) for emission into the upper half space. Vertical dotted, dashed, and solid black curves indicate the SMM result for the CEP-dependent cutoff energy. The grey horizontal line marks the critical phase predicted by M^3^C. (**c**) Evolution of the linear (Mie solution) and full M^3^C near field at the outer surface for the critical phase and sampled under the critical angle; all other parameters are the same as in (**a**). (**d**) Radially resolved evolution of the nonlinear near-field for the same parameters as in (**c**) and critical radial trajectories from SMM and M^3^C (average over trajectories with *E*=*E*_c_±1%). (**e**) CEP-dependent distributions of birth and recollision times of the M^3^C trajectories with *E*>0.8*E*_c_, see blue box in (**b**), and their time averages (red lines); black lines show the SMM prediction for cutoff electrons; the M^3^C recollision time refers to the mean of re-entry and exit, see zoomed region of panel (**d**); the *x*=0 passage of the peak of the unperturbed field envelope defines the time reference.

For a realistic description we employed quasi-classical Mean-field Mie Monte-Carlo (M^3^C) simulations (see Methods) and compared the results with the measured data (Fig. 2). Both the CEP-dependent switching of the photoemission and the size-dependent critical emission angles are well reproduced. M^3^C energy spectra (Fig. 4a) show that single recollision electrons are dominant over direct and higher order recollisions for all energies and the cutoff energy by far exceeds the SMM prediction. For classifying M^3^C trajectories, we counted each electron re-entry as one recollision event even if several microscopic collisions are involved. Both the quenching of direct electrons (because of the local trapping potential^8^) and the enhanced acceleration reflect the importance of charge interaction, which is analysed in more detail below. The resulting global M^3^C cutoff momentum (red filled circle in Fig. 3) is close to the experimental result regarding both magnitude and direction, indicating that all major effects contributing to the electron acceleration have been captured by the model.

### Attosecond recollision dynamics

To characterize the attosecond dynamics, we analysed CEP-dependent M^3^C data for emission into the upper half space. Energy spectra for single recollision electrons (Fig. 4b) visualize the cutoff energy modulation underlying the switching effect. By timing analysis (Fig. 4e) we find that, irrespective of CEP, essentially all fast electrons are born by tunnelling within a narrow time interval (<300 as) in a single dominant half-cycle. The nonlinear near-field contribution (see short-term evolution in Fig. 4d) reveals strong dynamics and has a significant effect on the local radial field (Fig. 4c). In particular, the resulting trapping field quenches tunnel ionization, as discussed in more detail later. However, although the ionization dynamics and energetics are substantially changed in the M^3^C treatment, the excursion length (Fig. 4d) and average timing (Fig. 4e) of recollision trajectories remain similar to the SMM prediction, substantiating the applicability of the recollision picture. Altogether, the half-cycle selectivity, the smooth systematic shift of birth and recollision times with CEP, and the resulting pronounced high-energy signal modulation give evidence for robust attosecond control.

### Systematic comparison of experiment and theory

**Figure 5:**
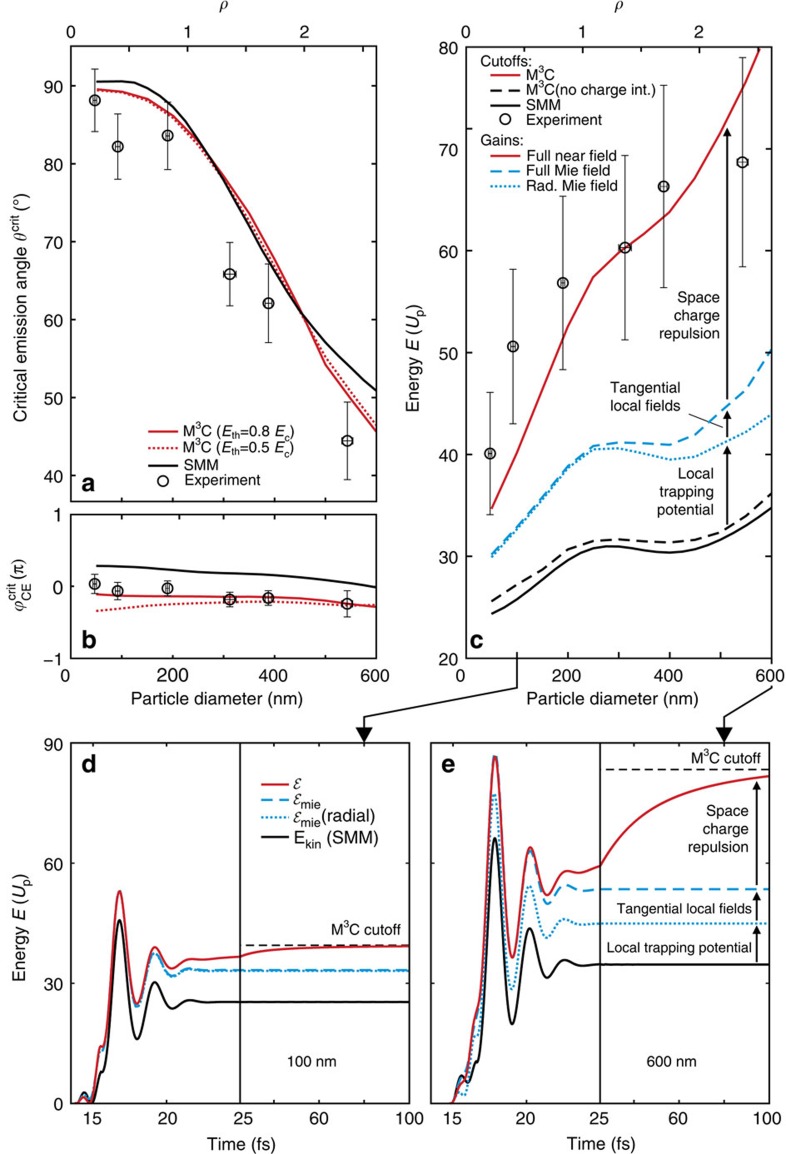
Size-dependent emission parameters. Comparison of measured (symbols) and calculated (lines) critical final emission angles 
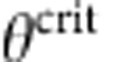
 (**a**), critical phases 
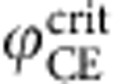
 (**b**), and cutoff energies *E*_c_ (**c**) as function of nanosphere size. Energies are given in units of the free space ponderomotive potential *U*_p_. The experimental and theoretical intensities are (3.0±0.5) × 10^13^ and 3.0 × 10^13^ W cm^−2^, respectively. Differences between solid and broken red lines in (**a**,**b**) indicate the small dependence of the M^3^C results on the threshold energy *E*_th_. M^3^C electron trajectories with *E*=*E*_c_±1% are used in (**c**) to calculate the selective energy gain 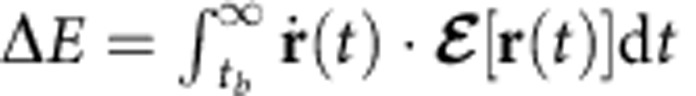 from different field contributions 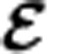. Compared with the SMM result, the energy gains calculated with Mie fields (blue lines) show the enhancement due to the local trapping potential. The difference between gains for radial and full Mie fields (dotted versus dashed blue line) quantifies the tangential field contribution. Space-charge repulsion is included when using the full self-consistent near-field for the energy gain integration, recovering the cutoff energy of the full M^3^C runs. For details see Methods. Note that because of low overall count rate for *d*=550 nm particles (low aerodynamic lens transmission) we might underestimate the cutoff energy for this size. The slope of the critical SMM phase in (**b**) mainly reflects the small trivial CEP shift, that is, the propagation induced CEP offset of the effective radial field relative to the incoming pulse. Horizontal error bars in (**a**–**c**) indicate the standard deviation of the particle diameter. Vertical error bars in (**a**) and (**b**) are composed by the standard deviations of upper and lower detector sides combined with systematic errors estimated to be 4° for 
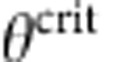
 and 0.1 *π* for 
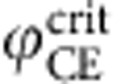
, respectively. The error for *E*_c_ in (**c**) is dominated by the uncertainty associated with laser intensity which is approximated to be ±15%. Uncertainties (≲5%) in the refractive index and work function because of surface contamination modify the simulation results by less than the experimental error bars. (**d**,**e**) Time evolution of the energy gains for two selected sphere sizes (as indicated); note the different scaling of the time axes before and after the black vertical lines. The pulse peak (in free space and at *x*=0) is set to *t*=15 fs.

The size-dependent impact of propagation-induced near-field deformation on the electron emission is analysed in Fig. 5. First, the evolution of measured critical angles (Fig. 5a) agrees well with both the SMM and M^3^C simulations, substantiating a systematically tunable directionality; the remaining offset between experiment and theory is attributed to a systematic error (for example, inhomogeneous VMI detector response). Second, the measured weakly size-dependent critical phase (Fig. 5b) shows a notable offset from the SMM result but is well described by M^3^C theory, supporting persistence of robust attosecond control and a proper description of the electron dynamics up to strongly deformed near-fields. Third, the measured cutoff energies are reasonably captured by the full M^3^C simulations but exceed the predictions of SMM and M^3^C theory with charge interaction switched off by up to a factor of two (Fig. 5c).

### Selective analysis of acceleration mechanisms

Finally, we disentangle and quantify the different many-particle contributions to the acceleration process using a selective energy gain analysis, see Methods for technical details. The resulting size-dependent analysis is depicted in Fig. 5c. The relative cutoff energy enhancement because of the local trapping potential is only weakly size-dependent and results mainly from the radial electron motion (compare black and blue lines). This behaviour supports that the trapping potential is only determined by the local electron density (surface charge density) and thus insensitive to the system size. The tangential field effect is notable only for large spheres (dashed versus solid blue line) but remains small even if tangential and radial field amplitudes become comparably strong, for example at *d*=500 nm (see Methods). This supports that the tangential field is neither crucial for the emission angles nor for the acceleration process. The relative enhancement due to the space-charge repulsion increases strongly with size and becomes dominant for large spheres. This trend can be explained with stronger Coulomb repulsion due to an increasing number of electrons in the escaping bunch, being an effect that is sensitive to the full (non-local) electron distribution.

The time-resolved analysis of the energy gain contributions in full M^3^C simulations shows the following dynamics, see examples in Fig. 5d,e. Compared with the SMM results (solid black curves), the enhancement of the gain associated with the radial Mie field (dotted blue curves) develops shortly after the moment of birth within the recollision process. The fact that no substantial change of the relative energy is found in later stages of the pulse supports that the impact of the trapping potential unfolds close to the surface. The gains calculated from the full Mie field (dashed blue curves) show that the tangential field effect also develops on short time-scales, that is, during the pulse. Finally, the energy gains with the full M^3^C field (red curves) reveal that the additional acceleration due to Coulomb explosion develops on a substantially longer timescale as it results solely from electron repulsion within the emitted bunch.

### Mean-field-induced quenching of the electron emission

**Figure 6:**
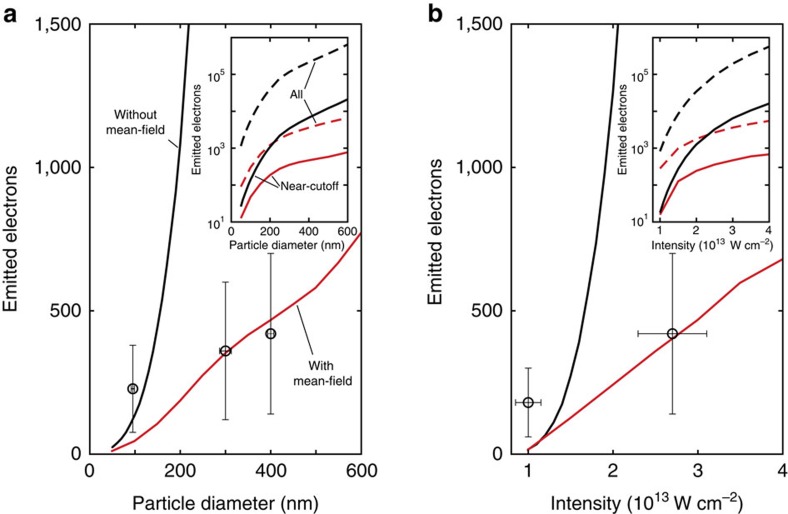
Size and intensity scaling of the electron yield. (**a**) Number of near-cutoff electrons emitted from a single nanosphere as function of laser intensity as predicted by M^3^C with (red curve) and without (black curve) Coulomb interaction and corresponding experimental results (circles) for *I*=3.0 × 10^13^ W cm^−2^. The inset compares the near-cutoff electron yield (solid curves) calculated with and without mean-field to the respective total yield (dashed curves)—note the logarithmic scale. Horizontal and vertical error bars indicate the standard deviation of the particle diameter, cf. Fig. 1, and the estimated detection efficiency (see text), respectively. (**b**) Same as (**a**) but as function of laser intensity for nanopsheres with *d*=400 nm. The experimental results were taken under comparable conditions. Note that only electrons with projected momenta above the threshold 
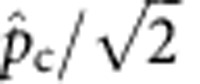
 where counted in the experiment. The horizontal error bar indicates the estimated ±15% uncertainty of the intensity; vertical error bars for the yield are defined as in (**a**). We checked via the M^3^C data that the differences between results using projected and full momenta are insignificant for this analysis.

In the investigated range of laser intensities and particle sizes we find a strong impact of the Coulomb field on the electron emission. The yield predicted by simulations without the Coulomb interaction scales roughly exponentially with sphere diameter and laser intensity, reflecting the highly nonlinear tunnelling rate. Inclusion of Coulomb effects decreases the yield by up to two orders of magnitude (red versus black dashed curves in the insets of Fig. 6a,b). The reduction is a direct consequence of the trapping field, which quenches tunnel ionization at the surface and limits the number of electrons that can escape with a given initial kinetic energy. This pivotal influence of the Coulomb field on the actual ionization dynamics also explains why the M^3^C results are only weakly affected from changes of the (certainly approximate) tunnelling rate (for example, by slight variations of the effective ionization potential) as soon as a substantial trapping field develops. On the other hand, the reasonable description of the Coulomb effects in the model then requires quantitative agreement of experiment and theory. However, a quantitative comparison of the total yield is difficult because of the spurious signal contribution from residual gas in our experiment and the imprint of focal averaging. To define a photoemission yield specific to nanoparticles and free from background gas contributions, we counted only electrons with momenta beyond the threshold 
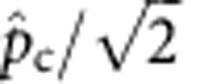
, which includes only signal well above the gas signal cutoff (see Fig. 1g). Further, focal averaging is circumvented by assigning most intense single-shot VMI images to the peak laser intensity. Because of the low particle density in the beam, such images reflect the emission from a single nanoparticle. Considering the estimated experimental electron detection efficiency of (30±20)%, the resulting measured near-cutoff electron yield as function of particle size and laser intensity is compatible with the corresponding M^3^C predictions with mean-field, see symbols and solid red lines in Fig. 6a,b. The remaining discrepancies for small particles and at low intensities are attributed to residual background signal. The agreement strongly supports the M^3^C prediction that the number of emitted electrons evolves nearly linearly with size and intensity in the presence of substantial Coulomb interaction.

## Discussion

The present results suggest that the size-dependent directionality of the strong-field photoemission from nanospheres directly relates to the field propagation-induced near-field deformation. The analysis reveals the dominance of the radial field driven recollision dynamics up to large sizes with substantial near-field ellipticity and gives evidence for the persistence of attosecond control of the electron dynamics. As the near-field deformation could in principle be manipulated directly via the excitation wavelength, our findings indicate feasibility of near-field-induced photoemission with optically tailored directionality with respect to the beam propagation axis.

Furthermore, we identify the transition from local-field-dominated dynamics^8^,^9^ to a regime where charge interaction effects due to the non-local structure of the emitted electron bunches become equally important or even dominant for the energetics of the electron acceleration. These results are of general relevance for strong-field electron dynamics in nanosystems (nanoparticles, -jets, -solids and -tips) and indicate the extension of near-field-mediated waveform control into the extremely nonlinear regime^21^,^22^. We anticipate that this enables steering of attosecond electron bunch emission from droplets^11^,^12^ via phase control and near-field enhancement of surface high-harmonic generation^23^ from nanostructured targets. Eventually, correlating the near-field driven electron dynamics with ion spectra^24^ and imaging the resulting particle damage via single-particle X-ray scattering promises unprecedented insights into the poorly understood processes of sub-wavelength laser machining in dielectrics^25^.

## Methods

### Sample preparation

Silica nanoparticles with diameters between 50 and 550 nm and a narrow size distribution were prepared by wet chemistry approaches. All chemicals (ethanol (Berkel AHK ultrapure, 100%), tetraethoxysilane (TES, Fluka, purum, 98%), ammonia solution (Merck, p.a., 28–30%)) were used as received without further purification. The reaction flasks were cleaned by hydrofluoric acid (8 vol.%) and ultrapure water before use. First, small seed nanoparticles were prepared by the Stöber method^26^. In a typical seed preparation procedure 35.41 g of TES and 43 ml of ammonia solution were added to 1,000 ml of ethanol and stirred for 12 h. After cleaning the sample by centrifugation and redispersion in ethanol, a further shell was grown on the silica nanoparticles by the seeded growth methods^27^,^28^ until the desired particle size between 50 and 550 nm was reached. In every step the dispersions were diluted to silica volume fractions of 0.5% and the ammonia and water concentrations were kept at 0.69 M NH_3_ and 1.56 M H_2_O, respectively. TES (30 ml (0.134 mol)) was added for each step. All samples have been stored in ultrapure ethanol or an ethanol ultrapure water mixture (80:20) after cleaning. Characterization by transmission electron microscopy and scanning electron microscopy yielded a polydispersity of ∼15% for small particles around 50 nm and decreases substantially to 3% for the large particles under study. The surface of silica nanoparticles prepared by the Stöber method are typically covered by silanols, that is, Si-OH groups^28^. Depending on the pH value of the surrounding environment the silanols can be protonated or deprotonated. At pH 7 the surface of silica nanoparticles is negatively charged and slightly hydrophilic^29^.

### Experimental approach

The few-cycle laser pulses were generated by spectral broadening of a Ti:Sa amplifier output (25 fs, 790 nm) using a Ne filled hollow-core fibre and chirped mirror compression. A fraction of the laser beam was split off and sent to a stereo atomic above-threshold ionization phasemeter^30^ for single-shot CEP measurement. The remaining laser beam was intersected with the nanoparticle beam in the focus of the VMI apparatus^9^,^31^. The jet of isolated nanospheres was generated from a dispersion of SiO_2_ particles in ethanol by aerodynamic lensing^32^. This technique ensured that the sample was refreshed between consecutive laser shots. The hit rate of nanoparticles was well below unity, ensuring single-particle conditions. Emitted electrons were projected onto a microchannel plate/phosphor screen assembly and recorded by a camera capable of acquiring single images at the laser repetition rate of 1 kHz. The VMI data was synchronized with the phase information from the phasemeter. In addition, laser shots containing no nanoparticle signal could be split off during analysis based on the number of electron events on the detector. No (Abel) inversion of the obtained momentum distributions was applied as the requirement of axial symmetry around the laser polarization axis is not met for larger nanoparticles. The background electron signal (mostly N_2_) of a nanoparticle scan served for laser parameter calibration (peak intensity, CEP offset) by comparison with separate scans in Xe.

### Linear few-cycle near-fields of nanospheres

For the time-domain description of the near-fields at the SiO_2_ nanospheres we employ Mie's continuous wave solution of the medium Maxwell equations combined with a spectral field decomposition. In linear response and spectral complex field representation, the spatiotemporal electric field evolution can be expressed as


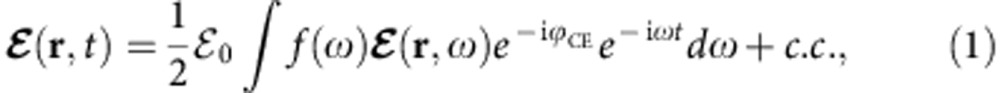


with 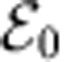 the field peak amplitude, *f*(*ω*) the normalized amplitude spectrum, 
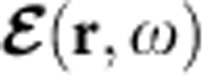
 the spatial mode structure as function of angular frequency, *ω*, and the CEP 
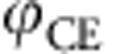
. For a bandwidth-limited few-cycle laser pulse (polarized along *y* axis, propagating along *x* axis) with Gaussian temporal field envelope, the spectrum and mode structure read


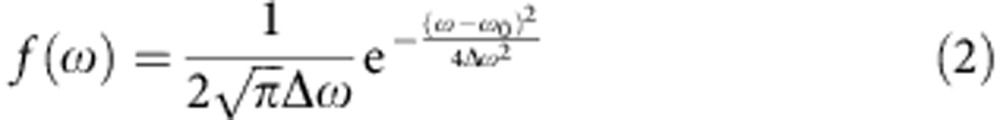



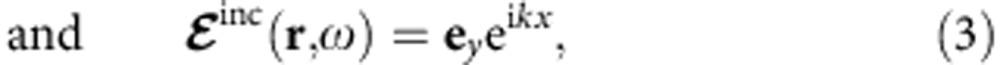


with 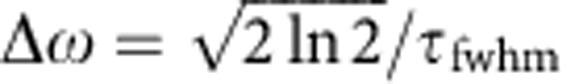, where *τ*_fwhm_ is the full-width at half-maximum of the temporal intensity envelope. The incident plane wave modes 
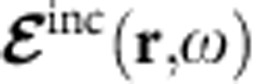
 provide the boundary conditions for the Helmholtz equation of a dielectric sphere and lead to the well-known Mie solutions


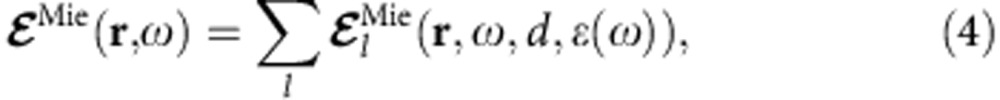


where the summation over *l* runs over the multipole orders of the expansion into spherical vector wave functions^33^ and *d* and *ɛ*(*ω*) describe the diameter and relative permittivity of the sphere. We evaluate the integral in equation (1) numerically by a discrete sum over 25 spectral components and by including multipole order up to *l*=5 in the Mie modes of equation (4). We checked that the dispersion of SiO_2_ is sufficiently small to justify the use of a fixed relative permittivity *ɛ*=2.12 sampled at the angular frequency *ω*_0_ corresponding to the laser central wavelength^34^.

### Near-field structure

**Figure 7:**
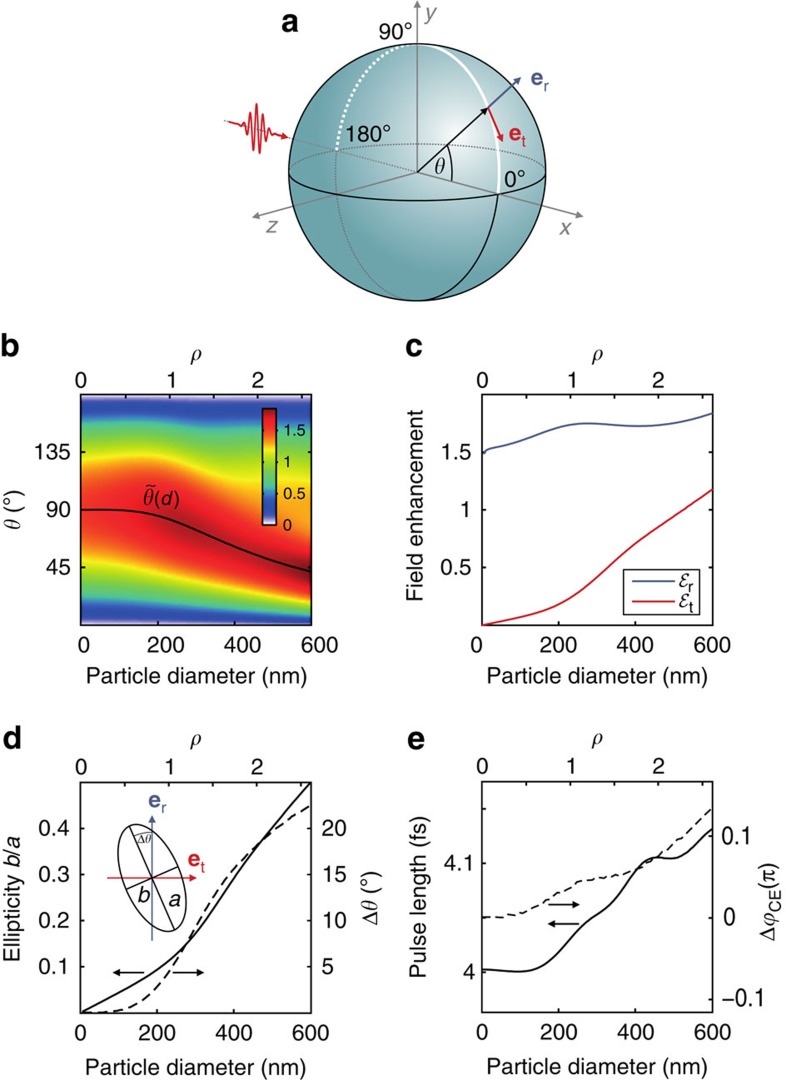
Size-dependent near-field enhancement. (**a**) Schematics of the polarization and propagation directions of the impinging few-cycle pulses and spatial trace at which the field is analysed (white arc). Radial and tangential fields (Mie solution) are evaluated by projection on the unit vectors ***e***_r_ and ***e***_t_ (as indicated). (**b**) Profiles of the local radial field enhancement, 
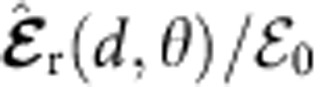
, and evolution of the characteristic angle, 
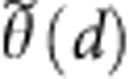
, with highest local radial field enhancement (black line). (**c**) Radial and tangential peak field amplitudes, 
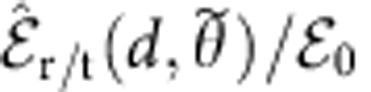
, sampled under the characteristic angle. (**d**) Field ellipticity (solid), defined via the ratio *b*/*a* of the field amplitudes along the minor and major field axis (see inset), and tilt angle Δ*θ* (dashed) of the major field axis with respect to the local surface normal (see inset). The unit vectors **e**_r_ and **e**_t_ in the inset are defined as in (**a**). (**e**) Effective pulse length (solid) and CEP shift 
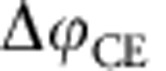
 (dashed) of the local radial field. Black arrows in (**d**) and (**e**) indicate the ordinate that correspond to each of the curves.

To characterize the size-dependent near-field we consider 4 fs pulses at 720 nm central wavelength and analyse the radial and tangential fields 
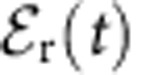
 and 
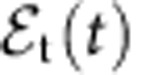
 in the *z*=0 plane at (outside) the nanosphere surface for *y*>0, see white arc in Fig. 7a, where the field component along the *z* axis vanishes for symmetry reasons. In the relevant size range, highest field enhancement occurs on this arc under the characteristic angle 
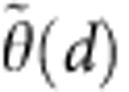
, see Fig. 7b. With increasing sphere size and under this characteristic angle, the radial and tangential peak amplitude show a modest and rapid increase, respectively (Fig. 7c). The resulting local fields become increasingly elliptic with size and exhibit a tilt of the major field axis with respect to the local surface normal (Fig. 7d). The pulse duration (intensity envelope) grows by <4% in the investigated size range and can thus be considered as essentially constant (Fig. 7e). Finally, the propagation effect introduces a small shift of the effective CEP of the radial field.

### Simulation model

In the quasi-classical M^3^C model, electron trajectories are generated via Monte-Carlo sampling of the surface tunnel ionization. In each time step, a tunnelling path is determined for randomly chosen surface atoms using an effective ionization potential of 9 eV to describe the band gap of SiO_2_ and following the local field direction. The ionization probability is determined from Ammosov–Delone–Krainov atomic tunnel ionization rates^35^ using the field gradient averaged over the tunnelling path. Trajectories are launched at the classical tunnel exit and integrated classically in the local electric fields. The latter contain the time-dependent Mie solution and the instantaneous, self-consistent mean-field. The mean-field describes the Coulomb interaction with free charges (electrons and residual ions) in the presence of the dielectric sphere via high-order multipole expansion up to multipole order *l*=10. In the mean-field solver, the sphere polarization is described by the same relative permittivity as in the Mie solver. Elastic electron–atom collisions are described by isotropic scattering events using an energy-dependent mean free path derived from quantum mechanical scattering cross-sections for the atomic potentials of Si and O atoms. Inelastic collisions are modelled with Lotz's electron impact ionization cross-sections^36^. Electron spectra for escaped electrons are calculated from trajectories with positive single-particle energy and positive normal velocity. A typical single high-resolution simulation run contains 2.5 × 10^6^ trajectories and takes ∼1 week on a single CPU core. The systematic scans over CEP and particle size performed in the current study required several tens of CPU years on the HLRN supercomputer, taking maximum advantage of massive parallel computation techniques. For the investigated laser intensities (*I*≲4 × 10^13^ W cm^−2^) adiabatic metallization^37^, breakdown effects^38^ and the nonlinear optical response of the dielectric sphere material^21^ can be neglected.

### Energy gain analysis

The contributions from the local trapping potential and space charge repulsion to the acceleration process are mediated by the self-consistent mean-field potential in the M^3^C simulations. To separate and quantify these effects we employ a selective energy gain analysis. Therefore, we consider the kinetic energy gain 
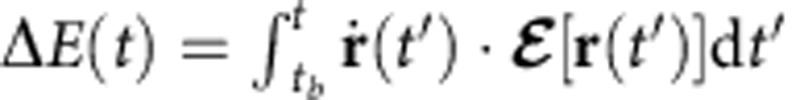
 resulting from different field contributions 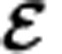. Considering the results of the SMM, evaluation of this integral for a given electron trajectory (and using only the Mie fields) is equal to the instantaneous kinetic energy. Likewise, integration over the full M^3^C trajectories and using the full near-field (Mie and dynamic mean-field) converges to the final asymptotic kinetic energy. For the analysis we select trajectories with final energy equal to the cutoff energy. To separate the effect of the local trapping potential, we use the full M^3^C trajectories but include only the Mie field in the energy gain integration. This includes the modification of the trajectories due to the presence of the mean-field, but excludes the acceleration mediated by its dynamical evolution, for example, due to Coulomb explosion of the escaping electron bunch.

## Additional information

**How to cite this article**: Süßmann, F. *et al.* Field propagation-induced directionality of carrier-envelope phase-controlled photoemission from nanospheres. *Nat. Commun.* 6:7944 doi: 10.1038/ncomms8944 (2015).

## References

[b1] Gramotnev D. K. & Bozhevolnyi S. I. Nanofocusing of electromagnetic radiation. Nat. Photon. 8, 13–22 (2014) .

[b2] Krausz F. & Stockman M. I. Attosecond metrology: from electron capture to future signal processing. Nat. Photon. 8, 205–213 (2014) .

[b3] Herink G., Solli D. R., Gulde M. & Ropers C. Field-driven photoemission from nanostructures quenches the quiver motion. Nature 483, 190–193 (2012) . 22398557 10.1038/nature10878

[b4] Piglosiewicz B. *et al.* Carrier-envelope phase effects on the strong-field photoemission of electrons from metallic nanostructures. Nat. Photon. 8, 37–42 (2014) .

[b5] Krüger M., Schenk M. & Hommelhoff P. Attosecond control of electrons emitted from a nanoscale metal tip. Nature 475, 78–81 (2011) . 21734706 10.1038/nature10196

[b6] Park D. J. *et al.* Characterizing the optical near-field in the vicinity of a sharp metallic nanoprobe by angle-resolved electron kinetic energy spectroscopy. Ann. Phys. 525, 135–142 (2013) .

[b7] Yanagisawa H. *et al.* Temporal and spectral disentanglement of laser-driven electron tunneling emission from a solid. Preprint at http://arxiv.org/abs/1405.0609v2 (2014) .

[b8] Zherebtsov S. *et al.* Controlled near-field enhanced electron acceleration from dielectric nanospheres with intense few-cycle laser fields. Nat. Phys. 7, 656–662 (2011) .

[b9] Zherebtsov S. *et al.* Carrier–envelope phase-tagged imaging of the controlled electron acceleration from SiO_2_ nanospheres in intense few-cycle laser fields. New J. Phys. 14, 075010 (2012) .

[b10] Dombi P. *et al.* Ultrafast Strong-Field photoemission from plasmonic nanoparticles. Nano Lett. 13, 674–678 (2013) . 23339740 10.1021/nl304365e PMC3573732

[b11] Liseykina T. V., Pirner S. & Bauer D. Relativistic attosecond electron bunches from laser-illuminated droplets. Phys. Rev. Lett. 104, 095002 (2010) . 20366990 10.1103/PhysRevLett.104.095002

[b12] Di Lucchio L. & Gibbon P. Relativistic attosecond electron bunch emission from few-cycle laser irradiated nanoscale droplets. Phys. Rev. ST Accel. Beams 18, 023402 (2015) .

[b13] Wollenhaupt M. & Baumert T. Ultrafast laser control of electron dynamics in atoms, molecules and solids. Farad. Discuss. 153, 9–26 (2011) . 10.1039/c1fd00109d 22452070

[b14] Lux C. *et al.* Circular dichroism in the photoelectron angular distributions of camphor and fenchone from multiphoton ionization with femtosecond laser pulses. Angew. Chem. Int. Ed. 51, 5001–5005 (2012) . 10.1002/anie.201109035 22351439

[b15] Mie G. Beiträge zur Optik trüber Medien, speziell kolloidaler Metallösungen. Ann. Phys 330, 377–445 (1908) .

[b16] Strutt J. On the scattering of light by small particle. Phil. Mag. 41, 447–454 (1871) .

[b17] Wang Z. *et al.* Optical virtual imaging at 50 nm lateral resolution with a white-light nanoscope. Nat. Commun. 2, 218 (2011) . 21364557 10.1038/ncomms1211

[b18] Peppernick S. J., Joly A. G., Beck K. M. & Hess W. P. Near-field focused photoemission from polystyrene microspheres studied with photoemission electron microscopy. J. Chem. Phys. 137, 014202 (2012) . 22779641 10.1063/1.4730598

[b19] Liou K., Takano Y. & Yang P. On geometric optics and surface waves for light scattering by spheres. J. Quant. Spectrosc. Radiat. Transf. 111, 1980–1989 (2010) .

[b20] Corkum P. B. Plasma perspective on strong-field multiphoton ionization. Phys. Rev. Lett. 71, 1994–1997 (1993) . 10054556 10.1103/PhysRevLett.71.1994

[b21] Schultze M. *et al.* Controlling dielectrics with the electric field of light. Nature 493, 75–78 (2013) . 23222519 10.1038/nature11720

[b22] Apalkov V. & Stockman M. I. Theory of dielectric nanofilms in strong ultrafast optical fields. Phys. Rev. B 86, 165118 (2012) .

[b23] Borot A. *et al.* Attosecond control of collective electron motion in plasmas. Nat. Phys 8, 416–421 (2012) .

[b24] Hickstein D. D. *et al.* Observation and control of shock waves in individual nanoplasmas. Phys. Rev. Lett. 112, 115004 (2014) . 24702383 10.1103/PhysRevLett.112.115004

[b25] Englert L. *et al.* Material processing of dielectrics with temporally asymmetric shaped femtosecond laser pulses on the nanometer scale. Appl. Phys. A 92, 749–753 (2008) .

[b26] Stöber W., Fink A. & Bohn E. Controlled growth of monodisperse silica spheres in the micron size range. J. Colloid Interf. Sci. 26, 62–69 (1968) .

[b27] Philipse A. P. Quantitative aspects of the growth of (charged) silica spheres. Colloid Polym. Sci. 266, 1174–1180 (1988) .

[b28] Van Blaaderen A., Van Geest J. & Vrij A. Monodisperse colloidal silica spheres from tetraalkoxysilanes: particle formation and growth mechanism. J. Colloid Interf. Sci. 154, 481–501 (1992) .

[b29] Cademartiri L. & Ozin G. Concepts of Nanochemistry Wiley (2009) .

[b30] Sayler A. M. *et al.* Precise, real-time, every-single-shot, carrier-envelope phase measurement of ultrashort laser pulses. Opt. Lett. 36, 1–3 (2011) . 21209667 10.1364/OL.36.000001

[b31] Süßmann F. *et al.* Single-shot velocity-map imaging of attosecond light-field control at kilohertz rate. Rev. Sci. Instrum 82, 093109 (2011) . 21974575 10.1063/1.3639333

[b32] Antonsson E. *et al.* Free nanoparticles studied by soft x-rays. Chem. Phys. Lett. 559, 1–11 (2013) .

[b33] Bohren C. F. & Huffman D. R. Absorption and Scattering of Light by Small Particles Wiley (1983) .

[b34] Khlebtsov B. N., Khanadeev V. A. & Khlebtsov N. G. Determination of the size, concentration, and refractive index of silica nanoparticles from turbidity spectra. Langmuir 24, 8964–8970 (2008) . 18590302 10.1021/la8010053

[b35] Ammosov M. V., Delone N. B. & Krainov V. P. Tunnel ionization of complex atoms and of atomic ions in an alternating electromagnetic field. Sov. Phys. JETP 64, 1191–1194 (1986) .

[b36] Lotz W. An empirical formula for the electron-impact ionization cross-section. Z. Phys. 206, 205–211 (1967) .

[b37] Durach M., Rusina A., Kling M. F. & Stockman M. I. Predicted ultrafast dynamic metallization of dielectric nanofilms by strong single-cycle optical fields. Phys. Rev. Lett. 107, 086602 (2011) . 21929186 10.1103/PhysRevLett.107.086602

[b38] Otobe T., Yabana K. & Iwata J.-I. First-principles calculation of the electron dynamics in crystalline SiO_2_. J. Phys. Condens. Matter 21, 064224 (2009) . 21715926 10.1088/0953-8984/21/6/064224

